# Efficacy of Weight Reduction on Pediatric Nonalcoholic Fatty Liver Disease: Opportunities to Improve Treatment Outcomes Through Pharmacotherapy

**DOI:** 10.3389/fendo.2021.663351

**Published:** 2021-04-13

**Authors:** Chance S. Friesen, Chelsea Hosey-Cojocari, Sherwin S. Chan, Iván L. Csanaky, Jonathan B. Wagner, Brooke R. Sweeney, Alec Friesen, Jason D. Fraser, Valentina Shakhnovich

**Affiliations:** ^1^ University of Kansas School of Medicine, Kansas City, KS, United States; ^2^ Children’s Mercy Kansas City, Kansas City, MO, United States; ^3^ University of Missouri-Kansas City School of Medicine, Kansas City, MO, United States; ^4^ University of Kansas Medical Center, Kansas City, KS, United States; ^5^ Center for Children’s Healthy Lifestyles & Nutrition, Kansas City, MO, United States

**Keywords:** NAFLD, pediatric, weight losing effect, pharmacotherapy, pediatric trials, pediatric NAFLD, weight loss efficacy

## Abstract

Obesity is the single greatest risk factor for nonalcoholic fatty liver disease (NAFLD). Without intervention, most pediatric patients with NAFLD continue to gain excessive weight, making early, effective weight loss intervention key for disease treatment and prevention of NAFLD progression. Unfortunately, outside of a closely monitored research setting, which is not representative of the real world, lifestyle modification success for weight loss in children is low. Bariatric surgery, though effective, is invasive and can worsen NAFLD postoperatively. Thus, there is an evolving and underutilized role for pharmacotherapy in children, both for weight reduction and NAFLD management. In this perspective article, we provide an overview of the efficacy of weight reduction on pediatric NAFLD treatment, discuss the pros and cons of currently approved pharmacotherapy options, as well as drugs commonly used off-label for weight reduction in children and adolescents. We also highlight gaps in, and opportunities for, streamlining obesity trials to include NAFLD assessment as a valuable, secondary, therapeutic outcome measure, which may aid drug repurposing. Finally, we describe the already available, and emerging, minimally-invasive biomarkers of NAFLD that could offer a safe and convenient alternative to liver biopsy in pediatric obesity and NAFLD trials.

## Introduction

With 340 million children worldwide affected by obesity (1), the rate of nonalcoholic fatty liver disease (NAFLD) is expected to rise because obesity remains the single most significant risk factor for NAFLD (2). The pathophysiology of NAFLD in obesity is complex and likely involves the interplay and dysregulation of multiple mechanisms beyond the scope of this publication (e.g., insulin resistance, cytokine signaling, gut microbiota) (3); however, continued abnormal weight gain is a common culprit in fueling the disease. NAFLD affects up to 70% of children and up to 90% of adults with obesity (2), and has become a leading indication for liver transplant in the United States (4). Without intervention, more than 75% of children with obesity will continue to gain excessive weight and become adults with obesity (5). Therefore, in addition to effective childhood weight management strategies, readily available non-invasive diagnostic and monitoring modalities are needed for NAFLD management, in order to prevent obesity-associated NAFLD progression from simple hepatic steatosis to steatohepatitis, fibrosis, cirrhosis, hepatocellular carcinoma, and end-stage liver failure in adulthood.

Weight reduction through lifestyle modification remains the first-line treatment approach for NAFLD (6). The present work aims to review the efficacy of weight reduction on pediatric NAFLD and to illustrate opportunities for improving NAFLD diagnosis, monitoring, and treatment outcomes through pharmacologic interventions.

## Lifestyle Modifications for Weight Reduction

**Figure 1:**
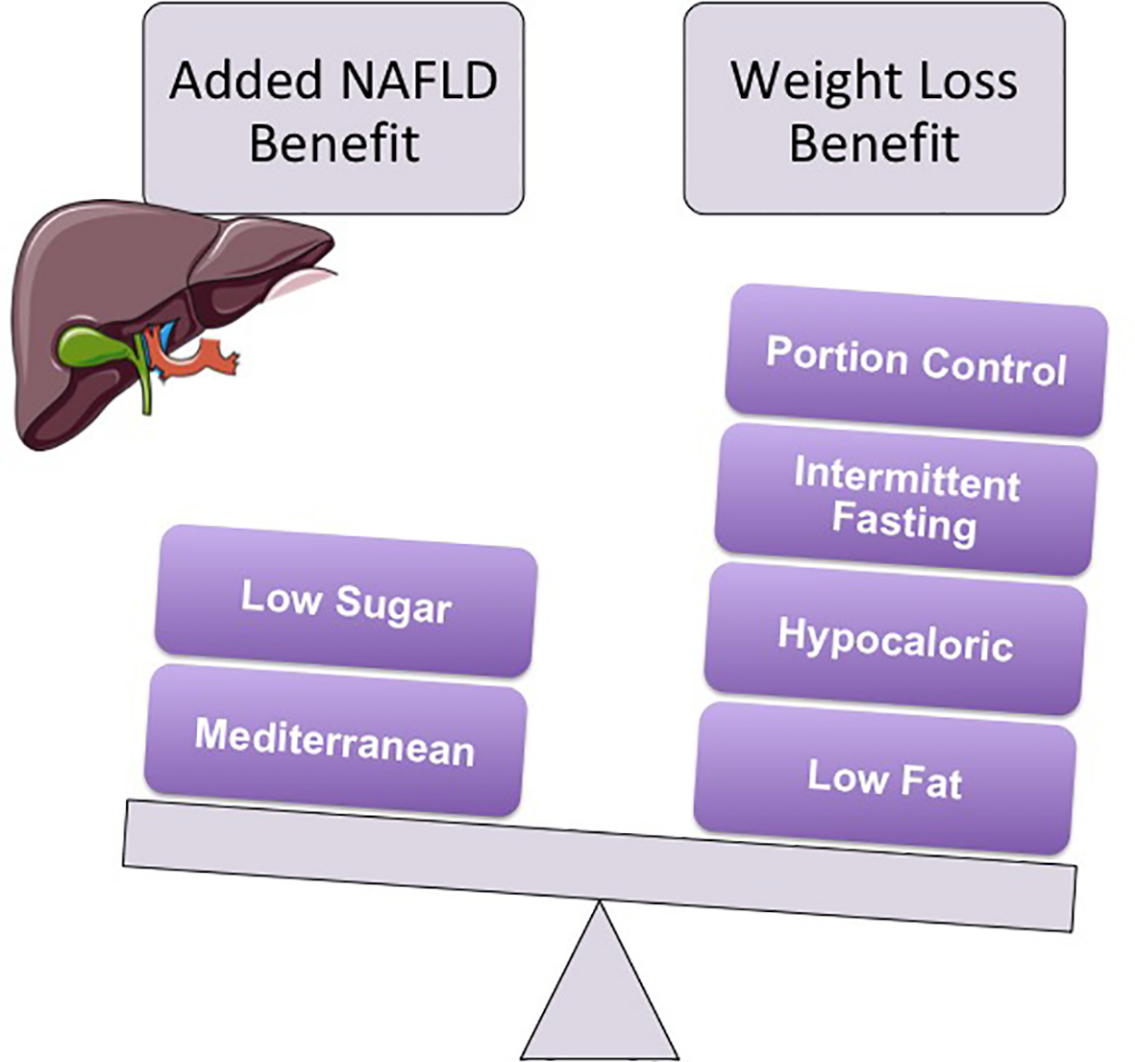
Multiple diets have shown efficacy for weight reduction in children. Ultimately, the best diet for weight management is the one that can be sustained over time. However, both the Mediterranean (i.e., low in red meat and high in fruits, vegetables, grains, nuts, grilled chicken and fish) and the low free sugar diet have shown added benefit in pediatric NAFLD, independent of weight reduction. Generally these diets are recommended in conjunction with regular exercise (e.g., at least 150 minutes/week of moderate intensity or >75 minutes/week of high intensity exercise), or moderate to vigorous physical activity daily for at least 20 minutes (with a target goal of 60 minutes daily).

Lifestyle modification for weight reduction and lasting improvement in health outcomes requires change in multiple areas, including diet and exercise (7). Weight loss of as little as one kilogram has been shown to improve clinical serum biomarkers of NAFLD in children (8), and biopsy-proven NAFLD if weight is subsequently maintained for 24 months (9). Consequently, many dietary interventions have been explored in pediatrics, including portion controlled, reduced glycemic load, low carbohydrate, meal replacement, and time restricted eating, as well as very low calorie diets and protein sparing modified fasting for more severe obesity (10–14). Similar to findings in adults (15), the most effective diet for pediatric weight management is the one that can be sustained successfully over time, with generally less adherence observed for low carbohydrate diets and better adherence to portion control and reduced glycemic load (10). These dietary changes are the recommended first step for NAFLD management, in combination with regular moderate to high intensity exercise (2, 6). Although there is currently insufficient evidence to recommend one type of exercise program over another for NAFLD (16), there is some evidence to suggest that a diet low in free sugar (17) and the Mediterranean diet specifically may have added benefit in treating NAFLD, independent of weight loss (18). Although adherence to these two diets was exceptionally high in some pediatric studies (8–10), this is atypical of most other research studies (19), and likely attributable to intense follow-up (8–10) and in-home interventions (10) that are not available, nor practical in the clinical setting. Dietary recommendations for weight loss and NAFLD are summarized in **Figure 1**, and are generally recommended in conjunction with exercise (>150 minutes/week moderate intensity, or >75 minutes/week high intensity) (16), or moderate to vigorous physical activity daily for at least 20 minutes (with a goal of 60 minutes) (7).

In clinical practice, these lifestyle modifications show heterogeneity in patient adherence and treatment response (20). In pediatrics, obesity is defined by body mass index (BMI) at or above the 95^th^ percentile for age and sex, and treatment response assessed using the change in the percent of the 95^th^ percentile (%BMIp95) (21, 22). In a large national prospective registry of children participating in non-surgical, multicomponent, intensive behavioral clinical treatment for obesity (n=6,454), the mean change in %BMIp95 after lifestyle modification was -1.88% at 4 to 6 months, -2.5% at 7 to 9 months, and -2.86% at >12 months – showing a modest decline in weight loss over time. Only those patients who achieved -5.2% change in %BMIp95 showed improvement in alanine aminotransferase (ALT), a clinical biomarker of liver injury (23). Whereas, those patients who experienced a change from normal to abnormal ALT had an increase in %BMIp95 of 0.48% (23), highlighting heterogeneity in treatment success.

Upon closer examination of the experience of a single program in this registry, only one third of the 769 participants achieved a >5% decrease in %BMIp95–the amount of BMI change associated with improvement in ALT and liver health (20). Increased number of clinical follow-up visits and days of physical activity were associated with greater odds of successful weight loss, defined as achieving >5% decrease in %BMIp95 (23). To illustrate the close clinical contact necessary for weight loss program success, guidance for moderately intense lifestyle modification therapy from the United States Preventative Services Task Force (USPSTF) recommends 26-75 patient contact hours over a 6-12 month period (24). However, in our experience, children with NAFLD are typically seen in clinic for approximately one hour every 2-3 months, falling short of the USPSTF-recommended contact hour target for weight management.

## Surgery for Weight Reduction

Bariatric surgery (e.g., laparoscopic sleeve gastrectomy) is an effective intervention for weight reduction (25), with post-surgical weight loss of approximately 20% total body weight in both adults (26) and children (27). Although less common in pediatrics, bariatric surgery is becoming an increasingly recognized option for children greater than 10 to 12 years of age with BMI >120% of the 95^th^ percentile (or BMI of 35 kg/m^2^) and comorbid NAFLD (26, 28). Recent investigations in adolescents suggest that bariatric surgery results in a four-fold reduction in hepatic fat fraction, with resolution of hepatic steatosis at 6 months post-surgery on imaging (29, 30), and 100% resolution of steatosis and 90% resolution of fibrosis on liver biopsy at 12 months post-surgery (27).

Despite its promise in treating pediatric obesity and NAFLD, bariatric surgery also appears to worsen NAFLD in a subset of patients. Although no pediatric data are available, in a large meta-analysis of 32 adult studies on bariatric surgery, 19 reported worsening or new NAFLD features, including steatosis, inflammation, and fibrosis in approximately 12% of patients postoperatively (25). Alarmingly, no apparent risk factors could be identified for this serious postoperative complication (25). Thus, the risks of bariatric surgery should be balanced against the potential for advantageous surgical outcomes for both obesity and NAFLD, and pharmacologic agents discussed as treatment adjuncts and/or alternative options.

## Pharmacotherapy for Weight Reduction

Currently, two pharmaceutical agents have approval from the United States Food and Drug Administration (FDA) for weight loss indications in children >12 years (orlistat and liraglutide), and one additional agent for adolescents >16 years (phentermine) (31, 32). Of these agents, orlistat’s use is somewhat limited by its gastrointestinal side-effect profile (e.g., diarrhea, bloating) (33); although, it was found to be moderately effective in decreasing BMI from 0.55 up to 4.2 kg/m^2^ (34–37). Phentermine is a stimulant and a Class IV controlled substance, necessitating increased consideration when prescribing in children. Additional screening and monitoring is necessary in children with congenital heart disease, cardiovascular disease, and thyroid abnormalities similar to protocols used when prescribing stimulants for other indications common in pediatrics (32). Phentermine also does not appear to be as effective in achieving weight loss in adolescents as in adults (31). As such, ligratulide–a GLP1 agonist newly approved by the FDA in December 2020 for the treatment of pediatric obesity–may soon emerge as the frontrunner. After somewhat disappointing BMI reduction results in an earlier trial in children with type II diabetes (38), results from a more recent, randomized, controlled trial demonstrate a decrease in BMI by 1.58 kg/m^2^ compared to placebo, when liraglutide was administered to children with obesity along with lifestyle modifications (39). Although therapeutic end-points for NAFLD were not included in this trial, greater than 5% reduction in BMI, previously associated with improvement in NAFLD (20), was achieved in almost half (43%) of liraglutide-treated participants, versus 19% of participants receiving lifestyle modification therapy alone (39). Additional GLP1 agonists are under investigation and it is our opinion that NAFLD assessment ought to be considered as a secondary treatment outcome in these trials, particularly as minimally-invasive biomarkers of NAFLD become more readily available and mitigate the need for liver biopsy.

The overall efficacy of GLP1 agonists appears comparable to other medications already labeled for pediatric indications and being used off-label for weight loss in children. For example, metformin, a first-line treatment for pediatric type II diabetes mellitus, has been associated with a modest BMI reduction in children (40). Recent studies looking at metformin demonstrated a decrease of -1.07 and -1.3 kg/m^2^ over 6 months (41), with a wide range of success (i.e., +0.38 to -2.52 kg/m^2^ BMI change) noted in some studies (40). Although the observed variability in treatment response may, in part, be related to variability in adherence to concomitant lifestyle modifications, we believe it is also related to variability in systemic drug exposure (i.e., pharmacokinetics) achieved from a given metformin dose administered to an individual child (42). Additional research is ongoing into potential sources of inter-individual variability (e.g., genetics) in metformin pharmacokinetics and pharmacodynamics (i.e., drug efficacy). Meanwhile, the GLP1 agonist exenatide appears to show efficacy comparable to metformin, with placebo-subtracted reduction of 1.3 kg/m^2^ (approximately -2.7% BMI) over 3 months, and -4% BMI over 6 months, in pediatrics (41). A small clinical study of topiramate, an anti-epilepsy drug already approved for pediatric use, also shows a 6-month decrease in BMI similar to exenatide (-4.6%), in 48 pediatric patients with severe obesity (43). Thus, medications currently indicated for pediatric weight reduction, as well as those approved for pediatric indications and administered off-label for the purpose of weight reduction, may have a role in weight management for pediatric patients. However, currently available data suggest that the range of weight loss achieved with pharmacotherapy may be only marginally greater than lifestyle modifications alone.

In randomized controlled trials, the mean weight change for lifestyle modification therapy was -1.8% BMI, compared to -2.8% BMI when therapy was combined with liraglutide or topiramate (44). Similarly, the mean pooled reduction in BMI in response to metformin therapy in children with obesity was -0.86 kg/m^2^, and fell within the BMI reduction ranges from lifestyle modifications alone (-0.02 to -1.10 kg/m^2^) (45). Importantly, on average, none of these weight management approaches achieved a reliable >5% reduction in BMI, deemed necessary for NAFLD improvement (20). Thus, though weight-reduction is an important first-line treatment for children with NAFLD, and may be achieved through lifestyle modifications, bariatric surgery or pharmacotherapy, each approach is associated with substantial variability in treatment response. This leaves a gap in universally effective treatments for pediatric NAFLD, and a potential role for precision therapeutics and pharmacotherapy.

## Pharmacotherapy for NAFLD

Currently, there are no FDA-approved medications for pediatric NAFLD. However, the landscape of potential therapeutic agents for NAFLD is evolving rapidly and showing promise, as highlighted in recently published reviews (26, 46) beyond the scope of this publication. In addition to novel therapeutic agents (e.g., obeticholic acid, fibroblast growth factor 19 & 21 analogs, thyroid hormone receptor-β agonists), there is also interest in repurposing existing drugs–already approved for pediatric obesity or obesity comorbidities–for the treatment of pediatric NAFLD (47).

One example is the lipid-lowering drug class statins. In addition to improving cardiovascular disease outcomes, statins have demonstrated significant improvement in hepatic function for patients with obesity and NAFLD (48, 49), likely through secondary anti-inflammatory effects. In adults with NAFLD, statin therapy is well-tolerated and has a similar, low frequency of hepatotoxicity comparable to placebo (48, 50), making it a potential candidate for NAFLD management. However, efficacy data remain equivocal (50) and genotype-stratified investigations may be necessary to identify therapeutic target ranges appropriate for NAFLD treatment indications.

It would be remiss not to mention oxidative stress as a potential therapeutic target in NAFLD management. Anti-oxidants (e.g. vitamin E, omega-3 fatty acids) have been used both for the treatment of pediatric weight reduction and NAFLD, with mixed results (8, 51–53). In one longitudinal, randomized, placebo-controlled trial, vitamin E did not demonstrate advantage over weight reduction through lifestyle modifications for NAFLD or obesity (8). In another longitudinal, placebo-controlled trial of vitamin E vs. metformin, neither agent resulted in sustained ALT reduction (51). However, resolution of steatohepatitis was significantly higher with vitamin E compared to placebo, even though BMI reduction was equivocal (51). Conversely, in a trial of omega-3 fatty acids, children treated with omega-3s were more likely to have BMI reduction compared to placebo, but reduction in ALT was equivocal (52, 53). Collectively, these studies indicate equipoise in anti-oxidant efficacy for NAFLD and highlight the need for further study.

Many other agents, already being used on and off-label for weight reduction, have been proposed to have therapeutic benefit for NAFLD (54), though few have been evaluated in children. These include orlistat; GLP1 agonists, such as liraglutide, semaglutide, and exenatide; and SGLT2 inhibitors. While some pediatric studies of orlistat have shown select improvement in serum liver injury markers (55), the majority of studies have not examined the effect of orlistat on liver adiposity or histopathology. Available data to date demonstrate no clear improvement in liver pathology that is independent of orlistat-induced weight loss (54), making orlistat a questionable drug candidate for primary NAFLD indications in children (56). To our knowledge, liraglutide and other GLP1 agonists have not been evaluated as pharmacotherapy for pediatric NAFLD; however, multiple studies in adults demonstrate promising improvement in hepatic steatosis and liver injury markers associated with GLP1 agonist treatment (54, 57). It is unclear whether this effect on NAFLD is independent of weight-loss, a caveat that extends to SGLT2 inhibitors, which also demonstrate some benefit for NAFLD treatment in adults (58). Parallel studies in pediatrics are indicated, particularly now that liraglutide is approved for weight loss indications in children, and more agents may be on the horizon soon.

Metformin, a drug already FDA-approved for type II diabetes in children and used off-label for pediatric weight loss (40, 41), has also shown some promise for repurposing in pediatric NAFLD. In a study of lifestyle modifications combined with either metformin or placebo in children with obesity and insulin resistance +/- NAFLD, the metformin group demonstrated a significant decrease in NAFLD scores and NAFLD prevalence, while the placebo group experienced an increase in both (59). Other studies in pediatrics using metformin at lower doses demonstrated isolated improvement in hepatic ballooning (51) or lobular inflammation (9) on histopathology, but failed to show an overall improvement in steatosis or NAFLD scores, suggesting that the effects of metformin on NAFLD may be dose-dependent. Improvement in serum ALT has been equivocal between studies, with 2 out of 4 pediatric studies demonstrating improvement in ALT (59, 60). However, this is likely because ALT, along with other currently available clinical markers of liver injury, lacks specificity for NAFLD (25).

## Minimally-Invasive Biomarkers of NAFLD

The diagnostic gold standard for NAFLD in children is liver biopsy. However, liver biopsy is invasive and not practical for recurrent monitoring of NAFLD. Instead, serum ALT is the most commonly used biomarker of NAFLD, but it is non-specific and can be normal in >80% of patients with NAFLD (25). Therefore, minimally-invasive and highly-specific biomarkers are urgently needed to diagnose and monitor NAFLD.

In the past decade, several new, potential biomarkers of NAFLD have been identified by serum proteomics/metabolomics analyses (61, 62). Based on their origin, these markers can be grouped into hepatokines, adipokines, and other cytokines. Although detailed review of these novel biomarker candidates is beyond the scope of this publication, the most promising biomarkers are summarized in **Table 1**. In addition, serum bile acids (BAs) are highlighted as another emerging class of biomarkers.

**Table 1:** Potential Biomarkers for Nonalcoholic Fatty Liver Disease.

Biomarkers & biomarker candidates	Type/origin	Changes in NAFLD steatosis	Correlations and changes in other NAFLD-related conditions	References
**Adiponectin**	Adipokine	↓	↓ in inflammation ↓ in fibrosis	(63)
**Chemerin**	Adipokine	↑	–	(64, 65)
**Leptin**	Adipokine	↑	↑ in inflammation ↑ in fibrosis	(66)
**Resistin**	Adipokine	( ↑ )	( ↑ ) in inflammation ( ↑ ) in fibrosis	(67)
**Visfatin**	Adipokine	↑	( ↑ ) in inflammation ( ↑ ) in fibrosis	(68, 69)
**Leptin/adiponectin ratio**	Adipokine	↑ NAFLD > obese children	positively correlates with triglyceride	(70)
**Irsin**	Adipokine	↑	( ↑ ) in inflammation ( ↑ ) in fibrosis	(71, 72)
**Fetuin- A, α2-HS-glycoprotein (AHSG)**	Hepatokine	↑	positively correlates with HOMA-IR (insulin resistance index)	(73, 74)
**Fibroblast growth factor 21 (FGF-21)**	Hepatokine	↑	positively correlates with hepatic fat fraction and hepatic triglycerides	(75–77)
**Selenoprotein P (SeP)**	Hepatokine	↑ in children ↓ in adults	negatively correlates with adiponectin	(77–79)
**Sex hormone-binding globulin (SHBG)**	Hepatokine	↓	–	(77)
**Leukocyte derived chemotaxin 2 (LECT2)**	Hepatokine	↑	↑ in insulin resistance	(80, 81)
**Cathepsin D (CatD)**	Hepatokine	↓	↓ in inflammation	(82)
**Adropin**	Hepatokine	↓	–	(83)
**Retinol-binding protein 4 (RBP4)**	Hepatokine, Adipokine	↓	↓ in insulin resistance & T2DM	(84)
**Tumor necrosis factor alpha (TNF-α)**	Inflammatory cytokine	↑	↑ in inflammation ↑ in fibrosis	(85, 86)
**Zonulin**	Intestinal peptide	↑ NAFLD > obese children	↑ in inflammation & NASH	(87, 88)
**Interleukin-18 (IL-18)**	cytokine	↑ NAFLD > obese children	–	(89)

↑ indicates marker increase; ↓ indicates marker decrease. NAFLD, nonalcoholic fatty liver disease; NASH, nonalcoholic steatohepatitis; T2DM, type II diabetes mellitus; HOMA-IR, homeostatic model assessment for insulin resistance.

BAs are synthesized in the liver, excreted into the bile, metabolized by the intestinal microbiome, and undergo several enterohepatic recirculations. Therefore, changes in the BA metabolome could be pathognomonic for various stages of NAFLD. The ratio of the primary BAs, cholic acid and chenodeoxycholic acid, is higher in patients with simple steatosis, as well as more advanced NAFLD (90). Compared to healthy controls, total fasting and postprandial primary BAs, and the ratios of taurine- and glycine-conjugated BAs, are consistently higher in the sera of adults with nonalcoholic steatohepatitis (NASH) (91). However, the role of secondary BAs as potential biomarkers remains debated, as the two studies above showed different results. Puri et al. observed lower total secondary BAs in adults with NASH compared to controls (90), whereas Ferslew et al. observed higher secondary BAs (91). In pediatrics, BAs study results are sparse and inconsistent, but could offer a valuable, minimally-invasive modality for NAFLD monitoring during treatment trials. Significantly lower total serum BA concentrations were found in one pediatric study of hepatic steatosis (92), but not in others (93, 94). Notably, two of these studies demonstrated decreased concentrations of glycine- and taurine-conjugated deoxycholic acid in children with NAFLD steatosis and/or NASH, compared to healthy controls (92, 93). The third study also found serum concentrations of glycine conjugated deoxycholic acid to be lower in children with NAFLD, but not in children with NASH (94). Together, these observations suggest that bile acid homeostasis in pediatric NAFLD may be distinct from the adult disease phenotype. Therefore, BAs results should not be extrapolated from adults to children, and further investigation in pediatric patients is warranted.

While serum biomarkers of NAFLD remain under investigation, several liver imaging biomarkers have become available. Magnetic resonance proton density fat fraction (MR-PDFF) is a non-invasive technique that quantifies the percentage of fat within a given volume of tissue. It correlates closely with steatosis grade on liver biopsy in children (95) and accurately captures longitudinal changes in pediatric NAFLD over time (96). However, it is only available in specialized tertiary care centers and is expensive. Ultrasound techniques for fat quantification are not yet widely available, but could offer a cheaper, more accessible alternative to magnetic resonance fat quantification, and facilitate more consistent and better quality disease monitoring for pediatric obesity and NAFLD trials.

For those imaging biomarkers that *are* widely available, one consistent shortcoming is that they perform best for tracking more severe stages of NAFLD/NASH, typical of adult disease. Grayscale ultrasound has been used for many years as a *qualitative* estimate of hepatic fat content. However, this technique is unreliable and non-specific for hepatic fat quantification, due to overlap in appearance between fatty and fibrotic liver (97, 98). Similarly, although both ultrasound and magnetic resonance elastography can quantify liver stiffness as a surrogate for fibrosis, NAFLD presents a challenge because measurement values are affected by both fat and fibrosis (99). Therefore, an ideal pediatric imaging surveillance program would incorporate *quantitative* imaging measurements that could discriminate fat and fibrosis.

## Discussion

Obesity is the single greatest risk factor for NAFLD in adults and children (2). Although weight loss of as little as 1 kilogram can improve NAFLD in children (8), consistent implementation and maintenance of intensive lifestyle modifications necessary to induce and sustain such weight loss are low (19, 20). Bariatric surgery is becoming increasingly recognized as an option for weight reduction in children (26), but it is invasive, and there is an unpredictable subset of patients who experience worsening liver fibrosis and NAFLD progression after surgery (25). This leaves room for pharmacologic interventions to aid with weight loss in pediatric obesity to treat and prevent NAFLD.

Without intervention, most pediatric patients with NAFLD continue to gain excessive weight (5) and become adults with NAFLD. Thus, inclusion of children and adolescents in pharmacology trials of novel weight-loss and NAFLD treatments is paramount. Repurposing drugs already labeled for pediatric use in comorbidities associated with obesity may be a complementary strategy to expand pediatric NAFLD treatment options beyond lifestyle modifications or bariatric surgery. Importantly, incorporation of NAFLD treatment end-points into obesity trials up-front would streamline efforts to assess a given drug’s efficacy for pediatric NAFLD, as well as obesity, and potentially help discriminate drug effect from weight-loss effect on NAFLD.

Regardless of the treatment approach selected, to streamline and facilitate treatment trials in pediatrics, minimally-invasive, highly-specific biomarkers of NAFLD are urgently needed to augment advanced imaging techniques that offer an alternative to liver biopsy, but are expensive and inaccessible in most clinical settings. A concerted effort must be made to include children in NAFLD biomarkers and therapeutics trials in order to intervene early and effectively in disease progression that is fueled by continued excessive weight gain from childhood into adulthood. Best strategies for including children in NAFLD trials are under development, as referenced in a draft guidance for industry from the FDA (100), and additional tips for optimal patient selection and age-appropriate therapeutic end-points can be found in a recent review by Alkhouri et al. (101).

## Data Availability Statement

The original contributions presented in the study are included in the article/supplementary material. Further inquiries can be directed to the corresponding author.

## Author Contributions

VS and CF conceived the idea for the manuscript. VS, CH-C, SC, IC, JW, BS, and JF provided expert opinion. All authors contributed to the article and approved the submitted version.

## Funding

This work was supported in part by grant 5K23DK115827-02 (V.S.), a CTSA grant from NCATS awarded to the University of Kansas for Frontiers: University of Kansas Clinical and Translational Science Institute (Ul1TR003266), with VS as a recipient, and a CTSA grant from NCATS awarded to the University of Kansas for Frontiers: University of Kansas Clinical and Translational Science Institute (KL2TR002367), with JW as recipient. The contents are solely the responsibility of the authors and do not necessarily represent the official views of the NIH or NCATS.

## Conflict of Interest

The authors declare that the research was conducted in the absence of any commercial or financial relationships that could be construed as a potential conflict of interest.

## References

[1] World Health Organization. (2018). Available at: https://www.who.int/end-childhood-obesity/publications/taking-action-childhood-obesity-report/en/ (Accessed January 11, 2021).

[2] Temple J Cordero P Li J Nguyen V Oben JA. A Guide to Non-Alcoholic Fatty Liver Disease in Childhood and Adolescence. Int J Mol Sci (2016) 17(6):947. 10.3390/ijms17060947 PMC4926480 27314342

[3] Fang YL Chen H Wang CL Liang L. Pathogenesis of non-alcoholic fatty liver disease in children and adolescence: From “two hit theory” to “multiple hit model”. World J Gastroenterol (2018) 24(27):2974–83. 10.3748/wjg.v24.i27.2974 PMC6054950 30038464

[4] Pais R Barritt A Calmus Y Scatton O Runge T Lebray P. NAFLD and liver transplantation: Current burden and expected challenges. J Hepatol (2016) 65(6):1245–57. 10.1016/j.jhep.2016.07.033 PMC5326676 27486010

[5] Lifshitz F. Obesity in Children. J Clin Res Pediatr Endocrinol (2008) 1(2):53–60. 10.4008/jcrpe.v1i2.35 21318065 PMC3005642

[6] Shah J Okubote T Alkhouri N. Overview of Updated Practice Guidelines for Pediatric Nonalcoholic Fatty Liver Disease. Gastroenterol Hepatol (2018) 14(7):407–14. PMC6111502 30166956

[7] Styne DM Arslanian SA Connor EL Farooqi IS Murad MH Silverstein JH. Pediatric Obesity-Assessment, Treatment, and Prevention: An Endocrine Society Clinical Practice Guideline. J Clin Endocrinol Metab (2017) 102(3):709–57. 10.1210/jc.2016-2573 PMC6283429 28359099

[8] Nobili V Manco M Devito R Ciampalini P Piemonte F Marcellini M. Effect of Vitamin E on Aminotransferase Levels and Insulin Resistance in Children with Nonalcoholic Fatty Liver Disease. Aliment Pharmacol Ther (2006) 24:1553–61. 10.1111/j.1365-2036.2006.03161.x 17206944

[9] Nobili V Manco M Devito R Di Ciommo V Comparcola D Sartorelli M. Lifestyle Intervention and Antioxidant Therapy in Children with Nonalcoholic Fatty Liver Disease: A Randomized, Controlled Trial. Hepatology (2008) 4(1):119–28. 10.1002/hep.22336 18537181

[10] Kirk S Brehm B Saelens BE Woo JG Kissel E D’Alessio D. Role of carbohydrate modification in weight management among obese children: a randomized clinical trial. J Pediatr (2012) 161(2):320–7. 10.1016/j.jpeds.2012.01.041 PMC3406261 22381024

[11] Berkowitz RI Wadden TA Gehrman CA Bishop-Gilyard CT Moore RH Womble LG. Meal replacements in the treatment of adolescent obesity: a randomized controlled trial. Obesity (Silver Spring) (2011) 19(6):1193–9. 10.1038/oby.2010.288 PMC3102147 21151016

[12] Fox CD Kaizer AM Rudser KD Nathan BM Gross AC Sunni M. Meal-Replacements followed by Topiramate for the Treatment of Adolescent Severe Obesity: A Pilot Randomized Controlled Trial. Obesity (Silver Spring) (2016) 24(12):2553–61. 10.1002/oby.21633 PMC5125846 27807925

[13] Jebeile H Gow ML Lister NB Haghighi MM Ayer J Cowell CT. Intermittent Energy Restriction Is a Feasible, Effective, and Acceptable Intervention to Treat Adolescents with Obesity. J Nutr (2019) 149(7):1189–97. 10.1093/jn/nxz049 31006807

[14] Andela S Burrows TL Baur LA Coyle DH Collins CE Gow ML. Efficacy of very low-energy diet programs for weight loss: A systematic review with meta-analysis of intervention studies in children and adolescents with obesity. Obes Rev (2019) 20(6):871–82. 10.1111/obr.12830 30734459

[15] Ge L Sadeghirad B Ball GDC da Costa BR Hitchcock CL Svendrovski A. Comparison of dietary macronutrient patterns of 14 popular named dietary programs for weight and cardiovascular risk factor reduction in adults: systematic review and network meta-analysis of randomized trials. BMJ (2020) 369:m696. 10.1136/bmj.m696 32238384 PMC7190064

[16] Schwimmer JB Ugalde-Nicalo P Welsh J Angeles J Cordero M Harlow K. Effect of a Low Free Sugar Diet vs Usual Diet on Nonalcoholic Fatty Liver Disease in Adolescent Boys. JAMA (2019) 321(3):256. 10.1001/jama.2018.20579 30667502 PMC6440226

[17] Thyfault JP Rector RS. Exercise Combats Hepatic Steatosis: Potential Mechanisms and Clinical Implications. Diabetes (2020) 69:517–24. 10.2337/dbi18-0043 PMC7085252 32198195

[18] Ryan MC Itsiopoulos C Thodis T Ward G Trost N Jofferberth S. The Mediterranean diet improves hepatic steatosis and insulin sensitivity in individuals with non-alcoholic fatty liver disease. J Hepatol (2013) 59(1):138–43. 10.1016/j.jhep.2013.02.012 23485520

[19] Cakir M Akbulut UE Okten A. Association between Adherence to the Mediterranean Diet and Presence of Nonalcoholic Fatty Liver Disease in Children. Child Obes (2016) 12(4):278–85. 10.1089/chi.2015.0197 26871614

[20] Gorecki MC Feinglass JM Binns HJ. Characteristics Associated with Successful Weight Management in Youth with Obesity. J Pediatr (2019) 212:35–43. 10.1016/j.jpeds.2019.05.039 31230887

[21] Freedman DS Berenson GS. Tracking of BMI z Scores for Severe Obesity. Pediatrics (2017) 140(3):e20171072. 10.1542/peds.2017-107 28830920 PMC5574726

[22] Freedman DS Butte NF Taveras EM Lundeen EA Blanck HM Goodman AB. BMI *z*-Scores are a poor indicator of adiposity among 2- to 19-year-olds with very high BMIs, NHANES 1999-2000 to 2013-2014. Obesity (2017) 25(4):739–46. 10.1002/oby.21782 PMC5373980 28245098

[23] Kumar S King EC Christison AL Kelly AS Ariza AJ Borzutzky C. Health Outcomes of Youth in Clinical Pediatric Weight Management Programs in POWER. J Pediatr (2019) 208:57–65.e4. 10.1016/j.jpeds.2018.12.049 30853195

[24] Whitlock EP O’Connor EA Williams SB Beil TL Lutz KW. Effectiveness of weight management interventions in children: a targeted systematic review for the USPSTF. Pediatrics (2010) 125(2):e396–418. 10.1542/peds.2009-1955 20083531

[25] Lee Y Doumouras AG Yu J Brar K Banfield L Gmora S. Complete Resolution of Nonalcoholic Fatty Liver Disease After Bariatric Surgery: A Systematic Review and Meta-analysis. Clin Gastroenterol Hepatol (2019) 17(6):1040–60. 10.1016/j.cgh.2018.10.017 30326299

[26] Nobili V Alisi A Valenti L Miele L Feldstein AE Alkhouri A. NAFLD in children: new genes, new diagnostic modalities and new drugs. Nat Rev Gastroenterol Hepatol (2019) 16:517–30. 10.1038/s41575-019-0169-z 31278377

[27] Manco M Mosca A De Peppo F Caccamo R Cutrere R Giordano U. The Benefit of Sleeve Gastrectomy in Obese Adolescents on Nonalcoholic Steatohepatitis and Hepatic Fibrosis. J Pediatr (2017) 180:31–7e2. 10.1016/j.jpeds.2016.08.101 27697327

[28] Pratt JSA Browne A Browne NT Bruzoni M Cohen M Desai A. ASMBS pediatric metabolic and bariatric surgery guidelines, 2018. Surg Obes Relat Dis (2018) 14:882–901. 10.1016/j.soard.2018.03.019 30077361 PMC6097871

[29] Luo RB Suzuki T Hooker JC Covarrubias Y Schlein A Liu S. How bariatric surgery affects liver volume and fat density in NAFLD patients. Surg Endosc (2018) 32:1675–82. 10.1007/s00464-017-5846-9 PMC6690434 29218660

[30] Hui SCN Wong SKH Ai Q Yeung DKW Ng EKW Chu WCW. Observed changes in brown, white, hepatic and pancreatic fat after bariatric surgery: evaluation with MRI. Eur Radiol (2019) 29(2):849–56. 10.1007/s00330-018-5611-z 30062524

[31] Ryder JR Kaizer A Rudser KD Gross A Kelly AS Fox CK. Effect of phentermine on weight reduction in a pediatric weight management clinic. Int J Obes (Lond) (2017) 41(1):90–3. 10.1038/ijo.2016.185 PMC5891125 27773937

[32] Srivastava G Fox CK Kelly AS Jastreboff AM Browne AF Browne NT. Clinical Considerations Regarding the Use of Obesity Pharmacotherapy in Adolescents with Obesity. Obesity (Silver Spring) (2019) 27(2):190– 204. 10.1002/oby.22385 30677262 PMC6449849

[33] Ozkan B Bereket A Turan S Keskin S. Addition of orlistat to conventional treatment in adolescents with severe obesity. Eur J Pediatr (2004) 163(12):738–41. 10.1007/s00431-004-1534-6 15378354

[34] O’ConnorEAEvansCVBurdaBUWalshESEderMLozanoP. Screening for Obesity and Intervention for Weight Management in Children and Adolescents: Evidence Report and Systematic Review for the US Preventive Services Task Force. JAMA (2017) 317(23):2427–44. 10.1001/jama.2017.0332 28632873

[35] McDuffie JR Calis KA Uwaifo GI Sebring NC Fallon EM Hubbard VS. Three-month tolerability of orlistat in adolescents with obesity-related comorbid conditions. Obes Res (2002) 10(7):642–50. 10.1038/oby.2002.87 12105286

[36] Chanoine JP Hampl S Jensen C Boldrin M Hauptman J. Effect of orlistat on weight and body composition in obese adolescents: a randomized controlled trial. JAMA (2005) 293(23):2873–83. 10.1001/jama.293.23.2873 15956632

[37] Maahs D de Serna DG Kolotkin RL Ralston S Sandate J Qualls C. Randomized, double-blind, placebo-controlled trial of orlistat for weight loss in adolescents. Endocr Pract (2006) 12(1):18–28. 10.4158/EP.12.1.18 16524859

[38] Danne T Biester T Kapitzke K Jacobsen SH Jacobsen LV Carlsson Petri KC. Liraglutide in an Adolescent Population with Obesity: A Randomized, Double-Blind, Placebo-Controlled 5-Week Trial to Assess Safety, Tolerability, and Pharmacokinetics of Liraglutide in Adolescents Aged 12-17 Years. J Pediatr (2017) 181:146–53.e143. 10.1016/j.jpeds.2016.10.076 27979579

[39] Kelly AS Auerbach P Barrientos-Perez M Gies I Hale PM Marcus C. A Randomized, Controlled Trial of Liraglutide for Adolescents with Obesity. N Engl J Med (2020) 382(22):2117–28. 10.1056/NEJMoa1916038 32233338

[40] McDonagh MS Selph S Ozpinar A Foley C. Systematic review of the benefits and risks of metformin in treating obesity in children aged 18 years and younger. JAMA Pediatr (2014) 168(2):178–84. 10.1001/jamapediatrics.2013.4200 24343296

[41] Kelly AS Fox CK Rudser KD Gross AC Ryder JR. Pediatric obesity pharmacotherapy: current state of the field, review of the literature and clinical trial considerations. Int J Obes (Lond) (2016) 40(7):1043–50. 10.1038/ijo.2016.69 PMC5546093 27113643

[42] Hosey-Cojocari C Halpin K Yan Y Leeder J. Towards an individualized dosing strategy to reduce pharmacokinetic (PK) variability and improve response to metformin in children with insulin resistance or type 2 diabetes. Clin Pharmacol Ther (2020) 107(Suppl 1):S110, Abstract PIII–117.

[43] Fox CK Marlatt KL Rudser KD Kelly AS. Topiramate for weight reduction in adolescents with severe obesity. Clin Pediatr (Phila) (2015) 54(1):19–24. 10.1177/0009922814542481 25027265 PMC5958908

[44] Ryder JR Kaizer AM Jenkins TM Kelly AS Inge TH Shaibi GQ. Heterogeneity in Response to Treatment of Adolescents with Severe Obesity: The Need for Precision Obesity Medicine. Obesity (Silver Spring) (2019) 27(2):288–94. 10.1002/oby.22369 PMC6352902 30677258

[45] O’ConnorEAEvansCVBurdaBUWalshESEderMLozanoP. Screening for Obesity and Intervention for Weight Management in Children and Adolescents Evidence Report and Systematic Review for the US Preventive Services Task Force. JAMA (2017) 317(23):2427–44. 10.1001/jama.2017.0332 28632873

[46] Attia SL Softic S Mouzaki M. Evolving Role for Pharmacotherapy in NAFLD/NASH. Clin Transl Sci (2021) 14(1):11–19. 10.1111/cts.12839 32583961 PMC7877845

[47] Friesen CS Chan SS Wagner JB Hosey-Cojocari C Csanaky IL Shakhnovich V. Critical Need For Pharmacologic Treatment Options In NAFLD: A Pediatric Perspective. Clin Transl Sci (2020) 00:1–3. 10.1111/cts.12952 PMC8212707 33476465

[48] Athyros VG Tziomalos K Gossios TD Griva T Anagnosstis P Kargiotis K. Safety and efficacy of long-term statin treatment for cardiovascular events in patients with coronary heart disease and abnormal liver tests in the Greek Atorvastatin and Coronary Heart Disease Evaluation (GREACE) Study: a post-hoc analysis. Lancet (2010) 376(9756):1916–22. 10.1016/S0140-6736(10)61272-X 21109302

[49] Tikkanen MJ Fayyad R Faergeman O Olsson AG Wun C Laskey R. Effect of intensive lipid lowering with atorvastatin on cardiovascular outcomes in coronary heart disease patients with mild-to-moderate baseline elevations in alanine aminotransferase levels. Int J Cardiol (2013) 168(4):3846–52. 10.1016/j.ijcard.2013.06.024 24001698

[50] Pastori D Polimeni L Baratta F Pani A Del Ben M Angelico F. The efficacy and safety of statins for the treatment of nonalcoholic fatty liver disease. Dig Liver Dis (2015) 47(1):4–11. 10.1016/j.dld.2014.07.170 25224698

[51] Lavine JE Schwimmer J Van Natta ML. Effect of Vitamin E or Metformin for Treatment of Nonalcoholic Fatty Liver Disease in Children and Adolescents: The TONIC Randomized Controlled Trial. JAMA (2011) 305:1659–68. 10.1001/jama.2011.520 PMC3110082 21521847

[52] Janczyk W Lebensztein D Wierzbicka-Rucinka A Mazur A Neuhoff-Murawska J Matusik P. Omega-3 Fatty acids therapy in children with nonalcoholic Fatty liver disease: a randomized controlled trial. J Pediatr (2015) 166(6):1358–63.e1-3. 10.1016/j.jpeds.2015.01.056 25771388

[53] Boyraz M Pirgon Ö Dündar B Çekmez F Hatipoğlu N. Long-Term Treatment with n-3 Polyunsaturated Fatty Acids as a Monotherapy in Children with Nonalcoholic Fatty Liver Disease. J Clin Res Pediatr Endocrinol (2015) 7(2):121–7. 10.4274/jcrpe.1749 PMC4563183 26316434

[54] Pan CS Stanley TL. Effect of Weight Loss Medications on Hepatic Steatosis and Steatohepatitis: A Systematic Review. Front Endocrinol (2020) 11:70. 10.3389/fendo.2020.00070 PMC7046622 32153507

[55] Norgren S Danielsson P Jurold R Lotborn M Marcus C. Orlistat treatment in obese prepubertal children: a pilot study. Acta Paediatr (2003) 92(6):666–70. 10.1111/j.1651-2227.2003.tb00596.x 12856974

[56] Mitchel EB Lavine JE. Review article: the management of paediatric nonalcoholic fatty liver disease. Aliment Pharmacol Ther (2014) 40(10):1155–70. 10.1111/apt.12972 25267322

[57] Mantovani A Petracca G Beatrice G Csermely A Lonardo A Targher G. Glucagon-Like Peptide-1 Receptor Agonists for Treatment of Nonalcoholic Fatty Liver Disease and Nonalcoholic Steatohepatitis: An Updated Meta-Analysis of Randomized Controlled Trials. Metabolites (2021) 11(2):73. 10.3390/metabo11020073 33513761 PMC7911747

[58] Scheen AJ. Beneficial effects of SGLT2 inhibitors on fatty liver in type 2 diabetes: A common comorbidity associated with severe complications. Diabetes Metab (2019) 45(3):213–23. 10.1016/j.diabet.2019.01.008 30708071

[59] Nadeau KJ Ehlers LB Zeitler PS Love-Osborne K. Treatment of Non-Alcoholic Fatty Liver Disease with Metformin versus Lifestyle Intervention in Insulin-Resistant Adolescents. Pediatr Diabetes (2009) 10(1):5–13. 10.1111/j.1399-5448.2008.00450.x 18721166

[60] Schwimmer JB Middleton MS Deutsch R Lavine JE. A phase 2 clinical trial of metformin as a treatment for non-diabetic paediatric non-alcoholic steatohepatitis. Aliment Pharmacol Ther (2005) 21(7):871–9. 10.1111/j.1365-2036.2005.02420.x 15801922

[61] Flisiak-Jackiewicz M Lebensztejn DM. Update on pathogenesis, diagnostics and therapy of nonalcoholic fatty liver disease in children. Clin Exp Hepatol (2019) 5:11–21. 10.5114/ceh.2019.83152 30915402 PMC6431091

[62] Miller MH Walsh SV Atrih A Huang JT Ferguson MA Dillon JF. Serum proteome of nonalcoholic fatty liver disease: A multimodal approach to discovery of biomarkers of nonalcoholic steatohepatitis. J Gastroenterol Hepatol (2014) 29:1839–47. 10.1111/jgh.12614 24750217

[63] Heydari M Cornide-Petronio ME Jimenez-Castro MB Peralta C. Data on adiponectin from 2010 to 2020: Therapeutic target and prognostic factor for liver diseases? Int J Mol Sci (2020) 21(15):5242. 10.3390/ijms21155242 PMC7432057 32718097

[64] Klusek-Oksiuta M Bialokoz-Kalinowska I Tarasow E Wojtkowska M Werpachowska I Lebensztejn DM. Chemerin as a novel non-invasive serum marker of intrahepatic lipid content in obese children. Ital J Pediatr (2014) 40:84. 10.1186/s13052-014-0084-4 25399407 PMC4237733

[65] Mohamed AA Sabry S Abdallah AM Elazeem NAA Refaey D Algebaly HAF. Circulating adipokines in children with nonalcoholic fatty liver disease: Possible noninvasive diagnostic markers. Ann Gastroenterol (2017) 30:457–63. 10.20524/aog.2017.0148 PMC5480001 28655985

[66] Polyzos SA Aronis KN Kountouras J Raptis DD Vasiloglou MF Mantzoros CS. Circulating leptin in non-alcoholic fatty liver disease: A systematic review and meta-analysis. Diabetologia (2016) 59:30–43. 10.1007/s00125-015-3769-3 26407715

[67] Senates E Colak Y Yesil A Coskunpinar E Sahin O Kahraman OT. Circulating resistin is elevated in patients with non-alcoholic fatty liver disease and is associated with steatosis, portal inflammation, insulin resistance and nonalcoholic steatohepatitis scores. Minerva Med (2012) 103:369–76. 23042372

[68] Johannsen K Flechtner-Mors M Kratzer W Koenig W Boehm BO Schmidberger J. Association between visfatin and hepatic steatosis in the general population during long-term follow-up. Horm Metab Res (2019) 51:602–7. 10.1055/a-0897-8565 31132798

[69] Elkabany ZA Hamza RT Ismail EAR Elsharkawy A Yosry A Musa S. Serum visfatin level as a noninvasive marker for nonalcoholic fatty liver disease in children and adolescents with obesity: Relation to transient elastography with controlled attenuation parameter. Eur J Gastroenterol Hepatol (2020) 32:1008–16. 10.1097/MEG.0000000000001608 31834057

[70] Angin Y Arslan N Kuralay F. Leptin-to-adiponectin ratio in obese adolescents with nonalcoholic fatty liver disease. Turk J Pediatr (2014) 56:259–66. 25341597

[71] Polyzos SA Kountouras J Anastasilakis AD Geladari EV Mantzoros CS. Irisin in patients with nonalcoholic fatty liver disease. Metabolism (2014) 63:207–17. 10.1016/j.metabol.2013.09.013 24140091

[72] Hu J Ke Y Wu F Liu S Ji C Zhu X. Circulating irisin levels in patients with nonalcoholic fatty liver disease: A systematic review and meta-analysis. Gastroenterol Res Pract (2020) 2020:8818191. 10.1155/2020/8818191 33224193 PMC7670588

[73] Reinehr T Roth CL. Fetuin-a and its relation to metabolic syndrome and fatty liver disease in obese children before and after weight loss. J Clin Endocrinol Metab (2008) 93:4479–85. 10.1210/jc.2008-1505 18728159

[74] Haukeland JW Dahl TB Yndestad A Gladhaug IP Loberg EM Haaland T. Fetuin a in nonalcoholic fatty liver disease: In vivo and in vitro studies. Eur J Endocrinol (2012) 166:503–10. 10.1530/EJE-11-0864 22170794

[75] Li H Fang Q Gao F Fan J Zhou J Wang X. Fibroblast growth factor 21 levels are increased in nonalcoholic fatty liver disease patients and are correlated with hepatic triglyceride. J Hepatol (2010) 53:934–40. 10.1016/j.jhep.2010.05.018 20675007

[76] Reinehr T Woelfle J Wunsch R Roth CL. Fibroblast growth factor 21 (fgf-21) and its relation to obesity, metabolic syndrome, and nonalcoholic fatty liver in children: A longitudinal analysis. J Clin Endocrinol Metab (2012) 97:2143–50. 10.1210/jc.2012-1221 22438225

[77] Flisiak-Jackiewicz M Bobrus-Chociej A Wasilewska N Tarasow E Wojtkowska M Lebensztejn DM. Can hepatokines be regarded as novel non-invasive serum biomarkers of intrahepatic lipid content in obese children? Adv Med Sci (2019) 64:280–84. 10.1016/j.advms.2019.02.005 30921653

[78] Choi HY Hwang SY Lee CH Hong HC Yang SJ Yoo HJ. Increased selenoprotein p levels in subjects with visceral obesity and nonalcoholic fatty liver disease. Diabetes Metab J (2013) 37:63–71. 10.4093/dmj.2013.37.1.63 23439771 PMC3579154

[79] Polyzos SA Kountouras J Tsatsoulis A Zafeiriadou E Katsiki E Patsiaoura K. Sex steroids and sex hormone-binding globulin in postmenopausal women with nonalcoholic fatty liver disease. Hormones (Athens) (2013) 12:405–16. 10.1007/BF03401306 24121382

[80] Lan F Misu H Chikamoto K Takayama H Kikuchi A Mohri K. Lect2 functions as a hepatokine that links obesity to skeletal muscle insulin resistance. Diabetes (2014) 63:1649–64. 10.2337/db13-0728 24478397

[81] Yoo HJ Hwang SY Choi JH Lee HJ Chung HS Seo JA. Association of leukocyte cell-derived chemotaxin 2 (lect2) with nafld, metabolic syndrome, and atherosclerosis. PloS One (2017) 12:e0174717. 10.1371/journal.pone.0174717 28376109 PMC5380318

[82] Walenbergh SM Houben T Hendrikx T Jeurissen ML van Gorp PJ Vreugdenhil AC. Plasma cathepsin d levels: A novel tool to predict pediatric hepatic inflammation. Am J Gastroenterol (2015) 110:462–70. 10.1038/ajg.2015.29 25732418

[83] Sayin O Tokgoz Y Arslan N. Investigation of adropin and leptin levels in pediatric obesity-related nonalcoholic fatty liver disease. J Pediatr Endocrinol Metab (2014) 27:479–84. 10.1515/jpem-2013-0296 24468600

[84] Romanowska A Lebensztejn DM Skiba E Tarasow E Kaczmarski M. Retinol binding protein-4 as a serum biomarker of intrahepatic lipid content in obese children–preliminary report. Acta Biochim Pol (2011) 58:35–8. 10.18388/abp.2011_2282 21383997

[85] Manco M Marcellini M Giannone G Nobili V. Correlation of serum tnf-alpha levels and histologic liver injury scores in pediatric nonalcoholic fatty liver disease. Am J Clin Pathol (2007) 127:954–60. 10.1309/6VJ4DWGYDU0XYJ8Q 17509993

[86] Jarrar MH Baranova A Collantes R Ranard B Stepanova M Bennett C. Adipokines and cytokines in non-alcoholic fatty liver disease. Aliment Pharmacol Ther (2008) 27:412–21. 10.1111/j.1365-2036.2007.03586.x 18081738

[87] Pacifico L Bonci E Marandola L Romaggioli S Bascetta S Chiesa C. Increased circulating zonulin in children with biopsy-proven nonalcoholic fatty liver disease. World J Gastroenterol (2014) 20:17107–14. 10.3748/wjg.v20.i45.17107 PMC4258579 25493023

[88] Hendy OM Elsabaawy MM Aref MM Khalaf FM Oda AMA El Shazly HM. Evaluation of circulating zonulin as a potential marker in the pathogenesis of nonalcoholic fatty liver disease. APMIS (2017) 125:607–13. 10.1111/apm.12696 28430371

[89] Flisiak-Jackiewicz M Bobrus-Chociej A Tarasow E Wojtkowska M Bialokoz-Kalinowska I Lebensztejn DM. Predictive role of interleukin-18 in liver steatosis in obese children. Can J Gastroenterol Hepatol (2018) 2018:3870454. 10.1155/2018/3870454 29854715 PMC5944203

[90] Puri P Daita K Joyce A Mirshahi F Santhekadur PK Cazanave S. The presence and severity of nonalcoholic steatohepatitis is associated with specific changes in circulating bile acids. Hepatology (2018) 67:534–48. 10.1002/hep.29359 PMC5764808 28696585

[91] Ferslew BC Xie G Johnston CK Su M Stewart PW Jia W. Altered bile acid metabolome in patients with nonalcoholic steatohepatitis. Dig Dis Sci (2015) 60:3318– 3328. 10.1007/s10620-015-3776-8 26138654 PMC4864493

[92] Jahnel J Zohrer E Alisi A Ferrari F Ceccarelli S De Vito R. Serum bile acid levels in children with nonalcoholic fatty liver disease. J Pediatr Gastroenterol Nutr (2015) 61:85–90. 10.1097/MPG.0000000000000774 25729888

[93] Lu LP Wan YP Xun PC Zhou KJ Chen C Cheng SY. Serum bile acid level and fatty acid composition in chinese children with nonalcoholic fatty liver disease. J Dig Dis (2017) 18:461–71. 10.1111/1751-2980.12494 28585279

[94] Nobili V Alisi A Mosca A Della Corte C Veraldi S De Vito R. Hepatic farnesoid x receptor protein level and circulating fibroblast growth factor 19 concentration in children with nafld. Liver Int (2018) 38:342–9. 10.1111/liv.13531 28746779

[95] Schwimmer JB Middleton MS Begling C Newton KP Awai HI Paiz MN. Magnetic resonance imaging and liver histology as biomarkers of hepatic steatosis in children with nonalcoholic fatty liver disease. Hepatology (2015) 61(6):1887–95. 10.1002/hep.27666 PMC4670559 25529941

[96] Middleton MS Van Natta ML Heba ER Alazraki A Trout AT Masand P. Diagnostic Accuracy of Magnetic Resonance Imaging. Hepatic Proton Density Fat Fraction in Pediatric Nonalcoholic Fatty Liver Disease. Hepatology (2018) 67(3):858–72. 10.1002/hep.29596 PMC6211296 29028128

[97] Expert Panel on Gastrointestinal Imaging Horowitz JM Arif-Tiwari H Asranis SK Hindman NM Kaur H. ACR Appropriateness Criteria® Chronic Liver Disease. J Am Coll Radiol (2017) 14(5S):S103–17. 10.1016/j.jacr.2017.02.011 28473066

[98] Tchelepi H Ralls PW Radin R Grant E. Sonography of diffuse liver disease. J Ultrasound Med (2002) 21(9):1023–32. 10.7863/jum.2002.21.9.1023 12216750

[99] Kim DW Park C Yoon HM Jung AY Lee JS Jung SC. Technical performance of shear wave elastography for measuring liver stiffness in pediatric and adolescent patients: a systematic review and meta-analysis. Eur Radiol (2019) 29(5):2560–72. 10.1007/s00330-018-5900-6 30617493

[100] United States Food and Drug Administration. (2018). Available at: https://www.fda.gov/media/119044/download (Accessed December 1, 2020).

[101] Alkhouri N Kohli R Feldstein AE. Designing Clinical Trials in Pediatric Nonalcoholic Steatohepatitis: Tips for Patient Selection and Appropriate Endpoints. Hepatol Commun (2019) 3(12):1563–70. 10.1002/hep4.1449 PMC6887671 31832567

